# Molecular Mechanism: Inhibition of *Fusarium oxysporum* T-2 Toxin Synthesis by Surfactin in Dried Fish: Induction of *Yap1* Nucleation by ROS Accumulation

**DOI:** 10.3390/molecules29225402

**Published:** 2024-11-15

**Authors:** Qi Deng, Xueting Ren, Qin Hu, Yuehua Pu, Lukman Iddrisu, Anand Kumar, Meifang Hua, Jianmeng Liao, Zhijia Fang, Ravi Gooneratne

**Affiliations:** 1 Guangdong Provincial Key Laboratory of Aquatic Product Processing and Safety, Guangdong Provincial Engineering Technology Research Center of Seafood, Guangdong Province Engineering Laboratory for Marine Biological Products, Key Laboratory of Advanced Processing of Aquatic Product of Guangdong Higher Education Institution, College of Food Science and Technology, Guangdong Ocean University, Zhanjiang 524088, China; dengqi@gdou.edu.cn (Q.D.); 2112203059@stu.gdou.edu.cn (X.R.); lukman@stu.gdou.edu.cn (L.I.); anandkumar@stu.gdou.edu.cn (A.K.); 2112003078@stu.gdou.edu.cn (M.H.); fangzhijia@gdou.edu.cn (Z.F.); 2 Collaborative Innovation Center of Seafood Deep Processing, Dalian Polytechnic University, Dalian 116034, China; 3 Development and Research Center for Biological Marine Resources, Southern Marine Science and Engineering Guangdong Laboratory (Zhanjiang), Zhanjiang 524002, China; 4 Zhanjiang Institute of Food and Drug Control, Zhanjiang 524022, China; puyuehua@gdsei.org.cn; 5 Guangdong Institute of Special Equipment Inspection and Research Zhanjiang Branch, Zhanjiang 524088, China; 6 Department of Wine, Food and Molecular Biosciences, Faculty of Agriculture and Life Sciences, Lincoln University, P.O. Box 85084, Lincoln 7647, New Zealand; ravi.gooneratne2@lincolnuni.ac.nz

**Keywords:** dried fish, surfactin, *F. oxysporum*, T-2 toxin, ROS, *Yap1*

## Abstract

(1) T-2 toxin synthesized by *Fusarium oxysporum* (*F. oxysporum*) can cause deterioration of dried fish and endanger human health. (2) The molecular mechanism by which antibacterial lipopeptides surfactin inhibited *F. oxysporum* growth and toxin production was elucidated by investigating the intracellular ROS production pathway and the subcellular distribution and transcriptional activity of the transcription factor *Yap1* and its regulation of *Tri5* gene in *F. oxysporum*. (3) Surfactin caused hyphal damage and nucleic acid and protein leakage; thus, the growth of *F. oxysporum* was disrupted. Surfactin’s excessive accumulation of intracellular ROS triggered the translocation of transcription factor *Yap1* into the nucleus, resulting in toxin cluster gene *Tri5* expression inhibition, thereby blocking T-2 toxin synthesis. (4) This is a novel mechanism by which surfactin inhibits the growth and T-2 toxin synthesis of *F. oxysporum* from multiple aspects, including cell structural integrity and the ROS-*Yap1* signaling pathway. (5) This study provides a theoretical basis for the application of surfactin in the antifungal control of aquatic dry products.

## 1. Introduction

Dried fish has a unique flavor and is rich in proteins, vitamins, and a variety of unsaturated fatty acids [1]. Dried fish play an important role in the economic development of coastal areas. However, dried fish are susceptible to fungal contamination during processing and storage [2], and *Fusarium oxysporum* (*F. oxysporum*) contamination is the main cause of mildew. Harm caused by *F. oxysporum* to fish and other dry aquatic products is mainly due to the high level of T-2 toxin produced by *F. oxysporum* in a low-biomass, high-protein, low-carbohydrate matrix, with the highest contamination reported being 1.03 mg/kg. This is well beyond the T-2 toxin safe limit (100 ng/kg) determined by the European Food Safety Authority (EFSA), which monitors food risks. T-2 is the most toxic A-type tricothecene family of compounds [3]. It is chemically stable and temperature-tolerant [4,5], and is difficult to destroy during the cooking and processing of dried fish. Long-term, low-dose consumption of dried fish products containing the T-2 toxin can affect human health [6,7,8]. Deng et al. [9] found that the amounts of T-2 toxin biosynthesis in dried red snapper and golden butterfish products sold in Zhanjiang, Guangdong Province, were 1.53 μg/kg and 0.86 μg/kg, respectively. These concentrations are too high, and therefore, this is an important issue to be addressed to improve dry aquatic product safety.

At present, prevention and control methods for mycotoxins include physical, chemical, and biological methods [10]. Physical and chemical methods have problems such as a short time limit, drug resistance, high cost, and flavor destruction [11]. Biological control methods are generally safe and cause less pollution. Among them, surfactin [12], a cyclic lipopeptide synthesized by *Bacillus subtilis*, is antibacterial and acts by damaging cell membranes [13]. Surfactin is non-toxic with no genotoxicity [14]. It significantly inhibits the growth of a variety of filamentous fungi, including *Aspergillus niger* and *Penicillium* [15,16]. Surfactin is potentially a biopreservative with strong surface activity and resistance to proteolytic degradation during salting and storage.

Surfactin inhibits mycotoxin biosynthesis, leading to the accumulation of reactive oxygen species (ROS) and activation of oxidative stress in fungal cells. As an important transcription factor in the fungal response to oxidative stress, yes-associated protein 1 (Yap1) can regulate toxin synthesis [17,18]. The impact of surfactin on *F. oxysporum* and its influence on growth and T-2 toxin biosynthesis by regulating key genes involved in redox signaling pathways has not been reported. The present study used surfactin to act on *F. oxysporum*, a high T-2 toxin producer in dried fish, and examined the role of *Yap1* in regulating T-2 toxin synthesis in *Tri5* in response to surfactin stress. This study aimed to elucidate the molecular mechanism by which surfactin blocks T-2 toxin synthesis in *F. oxysporum*. This was achieved by investigating the intracellular ROS production pathway in *F. oxysporum*, the subcellular distribution of the *Yap1* protein, and the regulatory effect on *Tri5* genes.

## 2. Results

### 2.1. Inhibitory Effect of Surfactin on the Growth Phenotype of F. oxysporum

**Figure 1:**
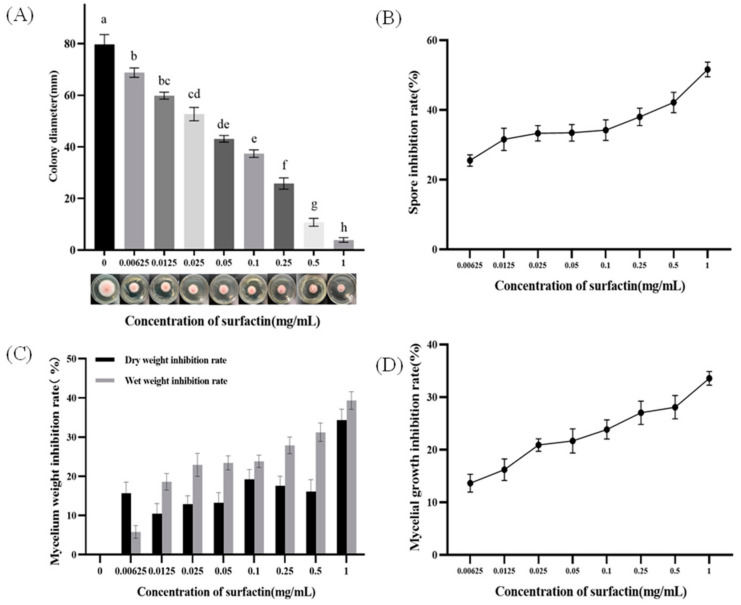
Inhibitory effect of surfactin on the growth phenotype of *F. oxysporum*. (**A**) illustrates the impact of varying surfactin concentrations on the colony diameter of *F. oxysporum* Fo17 on PDA plates following a 7-day incubation period. The inhibitory effect of surfactin on *F. oxysporum* spore production (**B**), biomass (**C**), and hyphal growth (**D**). In (**A**), the different letters represent significant differences between treatments.

The effects of surfactin on the colony diameter of *Fusarium spinosum* Fo17 are shown in Figure 1A. As the surfactin concentration increased, the colony diameter decreased significantly (*p* < 0.05), and the inhibitory effect of surfactin on the Fo17 colony positively correlated with the dose. The Fo17 growth was almost completely inhibited at 1 mg/mL surfactin concentration, indicating that this concentration can be used as the minimum inhibitory concentration (1 MIC) of surfactin on Fo17.

Spore production gradually declined with increasing surfactin concentration, whereas the spore inhibition rate of *F. oxysporum* demonstrated an upward trend. At a surfactin concentration of 0.00625 mg/mL, the spore inhibition rate was 25.1%, and the inhibition effect was deemed insignificant. At 1 mg/mL concentration, the spore production was the lowest, whereas the spore inhibition effect was the most pronounced, with an inhibition rate of 49.5%.

The increase in surfactin concentration positively correlated with the wet weight inhibition rate of mycelia. For instance, at surfactin concentrations of 0.00625 mg/mL and 0.0125 mg/mL, the wet weight inhibition rates were 8.3% and 10.5%, respectively, while at 1 mg/mL, the mycelia exhibited the lowest wet weight inhibition rate at 38.7%. Observation of the dry weight inhibition curves revealed that an inhibitory effect was evident even at low surfactin concentrations of 0.00625 mg/mL and 0.0125 mg/mL with dry weight inhibition rates of 2.23% and 18.6%, respectively. At a concentration of 1 mg/mL, the dry weight was the lowest, with significant inhibition of the *F. oxysporum* growth rate at 39.2%.

Figure 1D shows the mycelial growth inhibition of *F. oxysporum* at day 7 for different surfactant concentrations. As the surfactin concentration increased, the OD value decreased, indicative of gradual inhibition of *F. oxysporum* mycelial growth. The OD value was 0.545 at a 1 mg/mL surfactin concentration, significantly lower than the 0.821 OD value without surfactin, and the mycelial inhibition effect was the most pronounced.

### 2.2. Surfactin Induced Light and Scanning Electron Microscopic Changes in F. oxysporum Mycelia

**Figure 2:**
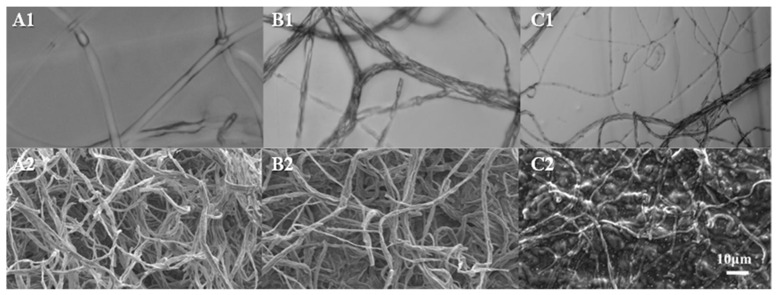
Effect of surfactin on the *F. oxysporum* (Fo17) hyphal structure. (**A1**–**C1**) show the light microscope morphology of Fo17 hyphae in the presence of 0, 0.5, and 1.0 mg/mL surfactin. (**A2**–**C2**) show scanning electron microscopic changes of Fo17 hyphae at 0, 0.5, and 1.0 mg/mL surfactin, respectively.

At a concentration of 0.5 mg/mL, the *F. oxysporum* mycelia exhibited tangling and thickening (Figure 2B). At a 1 mg/mL concentration, the mycelium, in addition, were twisted and appeared to break away to form strips of hyphae with an enlarged head (Figure 2C).

### 2.3. Effect of Surfactin on the Release of Nucleic Acids and Proteins from F. oxysporum

**Figure 3:**
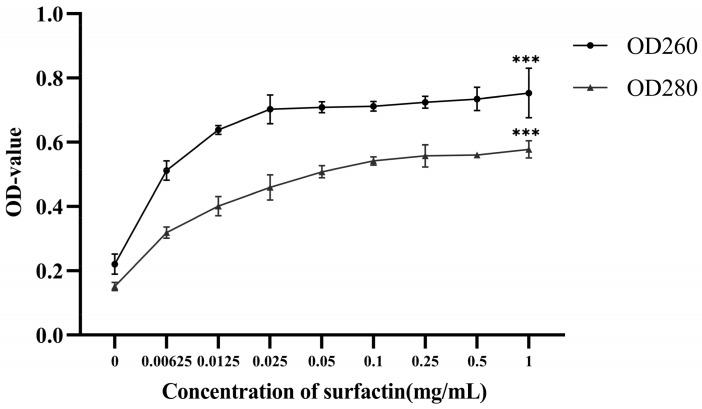
Effect of surfactin on *F. oxysporum* nucleic acid and protein release levels (***: *p* < 0.001).

Surfactin increased the nucleic acid and protein release by the *F. oxysporum* experimental group (Figure 3). The inhibitory effect of 1MIC surfactin on *F. oxysporum* growth was most pronounced with OD_260_ and OD_280_ values of 0.753 and 0.587, respectively. At 3 h post-treatment, the amounts of nucleic acids and proteins released were significantly higher (*p* < 0.001) than those in the control group. Thus, surfactin was an effective inhibitor of *F. oxysporum* growth. Surfactin disrupts the cell membrane of *F. oxysporum*, which subsequently leads to leakage of intracellular macromolecules.

### 2.4. Effect of Surfactin on the F. oxysporum ROS Production Pathway

**Figure 4:**
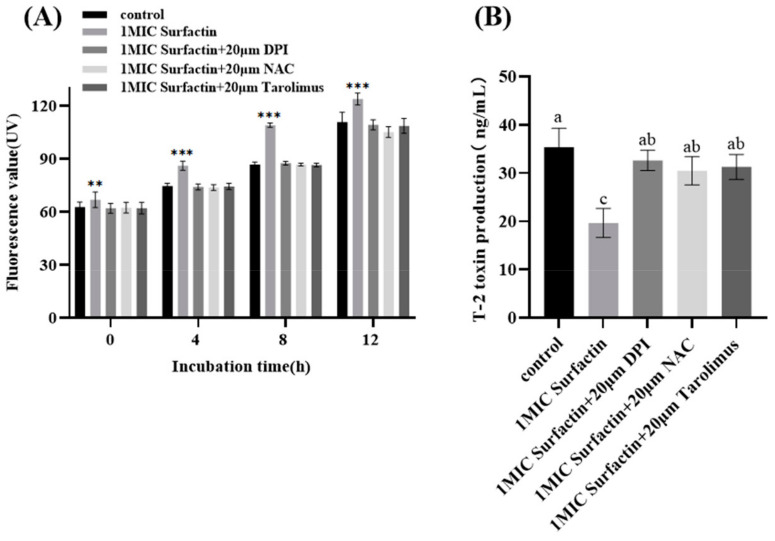
Inhibition of surfactin induced ROS accumulation (**A**) and T-2 toxin synthesis (**B**) in *F. oxysporum* by ROS inhibitors (**: *p* < 0.01; ***: *p* < 0.001). Different letters represent significant differences between treatments.

Reactive oxygen species (ROS) are reactive oxygen cluster groups produced in the mitochondria and endoplasmic reticulum during metabolism or in response to external stimuli. These factors regulate the growth and toxicity of *F. oxysporum*. The main known pathways that produce ROS are the calcium ion, mitogen-activated protein kinase (MAPK), and tyrosine kinase signaling pathways. One MIC addition of surfactin resulted in a significant elevation of intracellular *F. oxysporum* ROS levels compared to the control group (Figure 4A).

The addition of ROS inhibitors did not result in a significant increase in ROS levels. Furthermore, there was no significant difference between the three ROS inhibitors, indicating that 1 MIC surfactin has the capacity to significantly elevate intracellular (*p* < 0.01) ROS levels in *F. oxysporum* and that surfactin is not specific for the ROS production pathway. Addition of 1 MIC of surfactin resulted in a significant inhibition of T-2 toxin synthesis. The addition of three ROS inhibitors significantly attenuated the inhibitory effect of surfactin on T-2 toxin, resulting in a significant increase (*p* < 0.05) in T-2 toxin levels (Figure 4B). The absence of any significant difference in the attenuation by the three inhibitors suggests that the three ROS-generating pathways may act synergistically to regulate the T-2 toxin synthesis.

### 2.5. Critical Role of YRE Components in Yap1-Mediated Tri5 Response to Surfactin Stress

#### 2.5.1. Validation of *Yap1* Knockout Transformants

**Figure 5:**
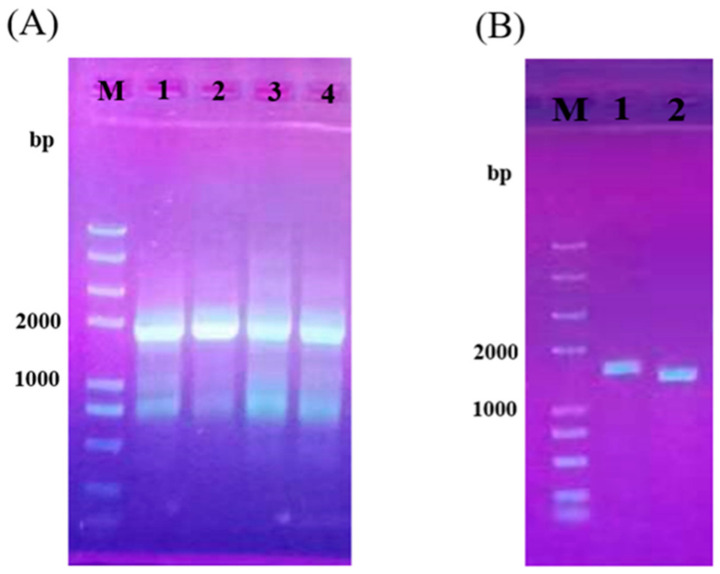
Amplified genes in *Yap1* knockout transformants. (**A**) shows the amplified genes HYG: M, DNA Markers; 1. Markers No. 1; 2. Invertor #2; 3. Invertor #3; 4. Invertor #4. (**B**) shows the amplified genes *Yap1*-5′-YY and *Yap1*-3′-YG: M, DNA markers; 1. Invertor #1 mycelial DNA was used as a template to amplify the *Yap1*-5′-HY and *Yap1*-3′-YG in *Yap1* knockdown transformants HY and *Yap1*-3′-YG: M is DNA Markers; 1. Invertor #1 mycelial DNA was used as a template to amplify *Yap1*-A1HY; 2. Invertor #1 mycelial DNA was used as template to amplify *Yap1*-A4YG).

The experiment validated the knockout transformants obtained from the *Yap1* knockout constructed using the knockout vector pBluescript KS (±) (pBS)-HPH1. Extracted mycelial DNA was used as a template to amplify the thiamphenicol resistance gene, HYG. The amplification demonstrated the presence of the expected size bands in all transformants numbered 1–4 (Figure 5A). The PCR products from transformant #1 were purified and sequenced (see Supplementary Materials: Sequencing splicing results of HYG gene amplification products). The mycelial DNA of transformant #1 was used as a template to amplify the *Yap1*-A1HY and *Yap1*-A4YG with primers *Yap1*-split-A1, HY, *Yap1*-A4, and YG. The amplified band sizes were consistent with the expected sizes (Figure 5B). The PCR products were purified and sequenced, and the sequencing results were correct (see Supplementary Materials: *Yap1*-5′-HY and ‘*Yap1*-3′-YG’ sequencing results and ‘*Yap1*-5′-HY and *Yap1*-3′-YG splicing sequence).

#### 2.5.2. The Phenotypic Alterations in the Defective Strain ∆*Yap1* (Deletion Strains of *Yap1)*

**Figure 6:**
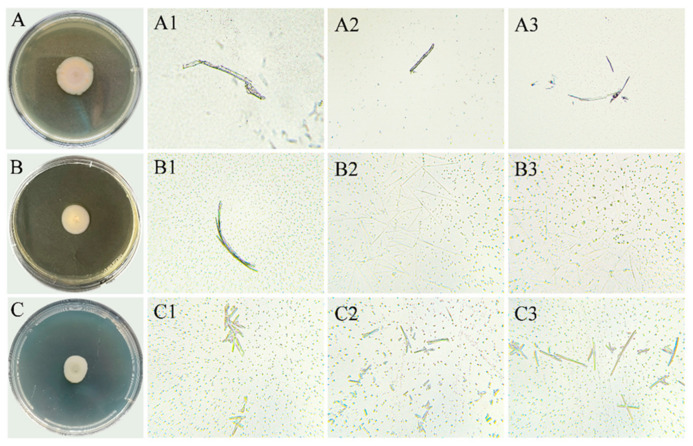
Colony and mycelial morphology of ∆*Yap1* cultured for 7 d in solid media. (**A**) = PDA; (**B**) = GYM; (**C**) = CMC; (**1**–**3**) show the structure of mycelium under a microscope (40×).

The morphology of the ∆*Yap1* strain exhibited notable differences from the wild type (Figure 6). These included a reduction in colony growth, an absence of aerial mycelia, and an absence of radial villi at the colony edge. The diameters of the colonies grown on potato agar culture PDA, GYM medium, and carboxymethylcellulose medium (CMC) were 23.58, 16.00, and 11.30 mm, respectively. The diameters of the colonies in the CMC were 23.58, 16.86, and 11.30 mm, respectively, following incubation in PDA, GYM medium, and CMC for 7 d. Microscopic observations revealed that the ∆*Yap1* strain exhibited enhanced sporulation ability in PDA, GYM, and CMC media, with an increased number of smaller spores. The mycelium were thin and long needle-shaped with fewer numbers and branches (Figure 6).

#### 2.5.3. Effect of *Yap1* Gene on *F. oxysporum* T-2 Toxin Production

**Figure 7:**
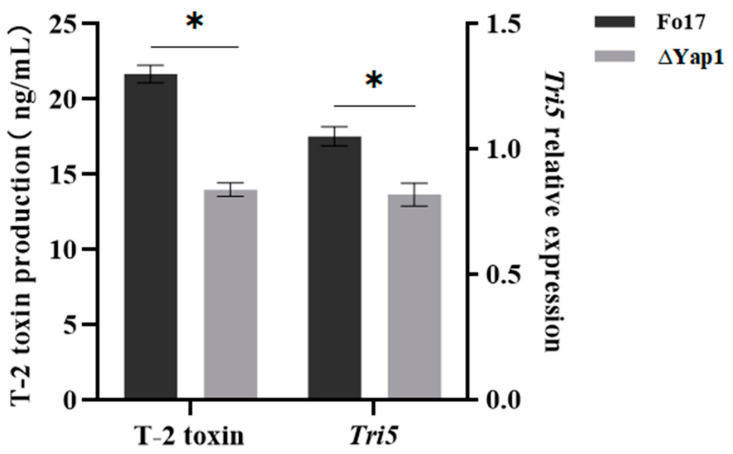
T-2 toxin synthesis and *Tri5* expression in Fo17 and ∆*Yap1* strains of *Fusarium oxysporum* (*: *p* < 0.05).

In the control group, the wild-type strain Fo17 produced 21.67 ng/mL T-2 toxin while the ∆*Yap1* strain synthesized only 13.98 ng/mL, indicating that the toxin-producing capability of the ∆*Yap1* strain is lower than that of the wild-type strain under comparable conditions (Figure 7). The expression of *Tri5*, the cluster gene for T-2 toxin synthesis, showed a correlation with T-2 toxin synthesis.

#### 2.5.4. Effect of Surfactin on the Subcellular Localization of the *Yap1* Gene in *F. oxysporum*

**Figure 8:**
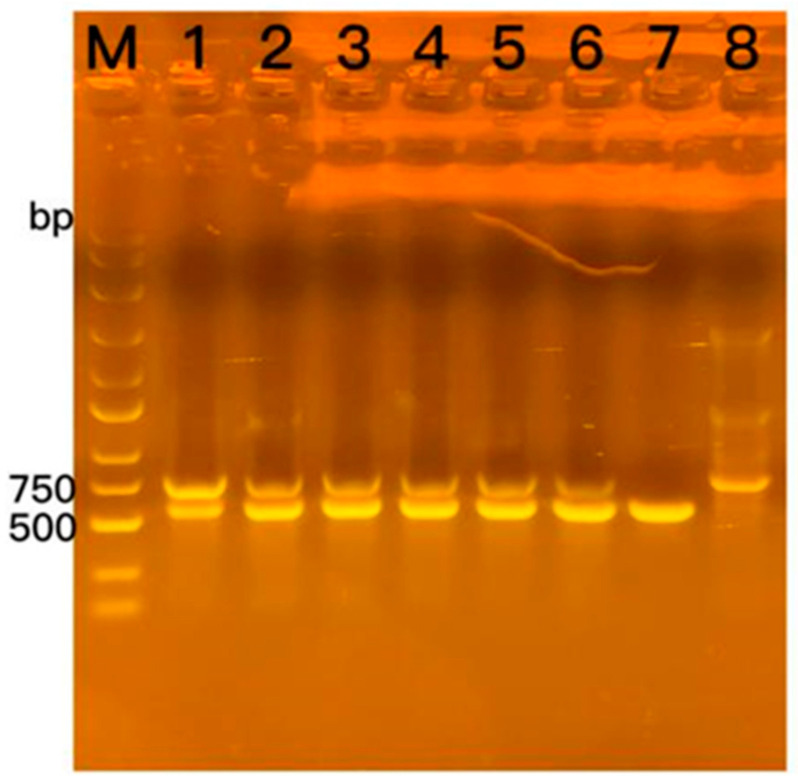
PCR validation of the backfill primers. M: 250 bp DNA ladder; 1: back-transformant Y1, 2: back-transformant Y2; 3: back-transformant Y3; 4: back-transformant G1; 5: back-transformant G2; 6: back-transformant G3; 7: 1300-neo-*Yap1* recombinant plasmid; 8: *F. oxysporum* (*Yap1*-deficient).

The *Yap1* portion of the gene, which was previously absent, was amplified using the backfill primer with the help of *Fusarium* transformant DNA and recombinant plasmid as templates. Furthermore, the recombinant plasmid exhibited a distinct band of >500 bp (Base Pair), whereas the *Yap1*-deficient *F. oxysporum* amplified with the backfill primer displayed a band size of 750 bp (Figure 8). The bright bands of the transformants of approximately 500 bp were excised and subjected to sequencing for verification.

**Figure 9:**
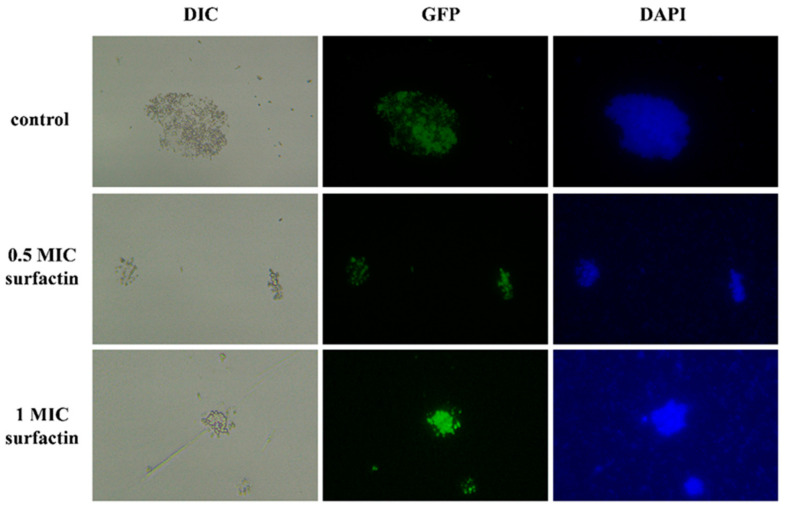
Effect of surfactin on the subcellular distribution of Fo17 *Yap1*.

As illustrated in Figure 9, green fluorescence was observed in the cytoplasm of the *Yap1*-GFP strain by fluorescence microscopy, indicating that *Yap1* was localized in the cytoplasm. Following surfactin treatment, the nucleus of the *Yap1*-GFP strain exhibited obvious green fluorescence, suggesting that surfactin treatment facilitated transfer of the *Yap1* protein from the cytoplasm to the nucleus, thereby regulating the expression of T-2 toxin-related genes. A comparison of the NCBI database revealed that the *Yap1* protein may recognize the YRE sequence as 5′-TGACTAA-3′ within the *Tri5* promoter region (https://www.ncbi.nlm.nih.gov/nuccore/AFNW01000623.1?report=fasta; accessed on 8 August 2024).

## 3. Discussion

To our knowledge, this is the first study on the effect of surfactin on the growth phenotype of *F. oxysporum* in dried fish. Surfactin significantly inhibited the growth (*p* < 0.01) and virulence of Fo17 (*p* < 0.01), and also significantly disrupted the *F. oxysporum* mycelium morphology, resulting in curvature, breakage, atrophy, and head-end enlargement of the mycelia. Surfactin interacts with the cell membrane lipid layer and affects membrane permeability regulation [19]. In this study, surfactin increased the Fo17 nucleic acid and protein leakage, which suggests that surfactin can alter the permeability of Fo17, the cell membrane, resulting in leakage of intracellular macromolecules and inhibition of cell growth and metabolism, ultimately leading to the fungal death. This is consistent with a previous report that [ΔLeu6] surfactin can cause mycelial disruption and cellular leakage of nucleic acids and proteins, which is consistent with the results of the present study [20]. However, in contrast to the results of the present study, [ΔLeu6] surfactin did not affect the reactive oxygen species (ROS) levels in *Candida albicans*.

External environmental factors have been implicated in the regulation of fungal morphological transformation and secondary metabolism. Oxidative stress plays a key role in these processes [21]. The biosynthesis of several fungal toxins (aflatoxin, zanthoxin, OTA, *Fusarium* toxin) is influenced by ROS [22,23]. In *Saccharomyces cerevisiae*, *Yap1* is involved in maintaining the intracellular redox homeostasis and activating oxidative response genes. In the present study, surfactin caused excessive accumulation of intracellular ROS in *Fusarium spinosum*, inducing oxidative stress, similarly to miconazole, which induces dose-dependent ROS production in *C. albicans* to generate antibacterial activity [24].

To investigate the mechanism by which the reactive oxygen transcription factor *Yap1*-mediated surfactin blocks T-2 toxin synthesis by *F. oxysporum* in dried fish, the target gene was knocked out and green fluorescently labeled to obtain a *Yap1* knockout strain (∆*Yap1*) and a backfill strain of *Yap1* (with a green fluorescent marker for GFP) (*Yap1*-GFP). Similarly, *Fusarium graminearum Yap1* gene deleted strains showed folds in the mycelia compared to the wild type, with a small increase in sporulation and a decrease in toxin production compared to the wild type [25]. This suggests that *Yap1* interacts with virulent genes to regulate the *F. oxysporum* virulence. In the inactivated state, the *Yap1* protein is predominantly found in the cytoplasm [26]. The external oxidative signal is first sensed by the intracellular stress protein ORP1/GPX3, which then transmits the signal to the Ybp1 and *Yap1* protein complex, which transforms reduced *Yap1* to the oxidized form, ultimately leading to a continuous accumulation of *Yap1* in the nucleus of the organism. This in turn regulates the expression of certain related genes in order to adapt to the changes in oxidative stress induced by the external environment [27,28,29]. In our study, the *Yap1* gene was green fluorescent protein (GFP)-labeled and backfilled (*Yap1*-GFP) for detection via fluorescence microscopy. Aggregated cytoplasmic *Yap1* moved to the nucleus. Thus, surfactin stress induced accumulation of ROS, which caused *Yap1* to aggregate in the nucleus, and the oxidative stress signal was transmitted to downstream toxin-encoding genes. *Yap1* recognized the YRE sequence of the *Tri5* promoter region in the nucleus to bind and form the *Yap1*-*Tri5* complex, resulting in inhibition and, hence, the expression of the *Tri5* gene to block the T-2 toxin synthesis.

## 4. Materials and Methods

### 4.1. Materials

Surfactin was purchased from Shanghai yuanye Bio-Technology Co., Ltd. (Shanghai, China), C_53_H_93_N_7_O_13_, CAS No. 24730-31-2. The T-2 toxin standard from Enzo Life Science (Farmingdale, New York, NY, USA) was used. Wild-type *Fusarium oxysporum* (Fo17, GDMCC60824), which has a strong capacity to synthesize the T-2 toxin, was isolated from dried fish (see Table S3 and Figure S1 in the Supplementary Materials).

### 4.2. Experimental Methods

#### 4.2.1. Inhibitory Effect of Surfactin on *F. oxysporum* Growth Phenotype

##### Minimum Inhibitory Concentration (MIC) of Surfactin on *F. oxysporum* (Fo17) Growth

The antifungal properties of surfactin were quantified by measuring the diameters of fungal colonies in Petri dishes [30]. A 1 L solution of Potato Dextrose Agar (PDA) medium was prepared, and surfactin was added to create 0.00625, 0.0125, 0.025, 0.05, 0.1, 0.25, 0.5, and 1 mg/mL concentrations. The control group, which did not receive surfactin, was used as a comparator. Fo17 strains, with diameters of 4 mm, were transferred to PDA plates and incubated at a constant temperature of 28 °C for 7 days. Photographs were taken to observe morphological changes in the colony.

##### Effect of Surfactin on the *F. oxysporum* (Fo17) Mycelial Biomass

A 2 mL sample of 8 × 10^6^ cfu/mL Fo17 suspension was introduced into 18 mL of GMS liquid medium. The mixture was incubated at 28 °C and 37 °C at a speed of 80 rotations per minute, and samples were taken at 24, 44, 48, 56, and 72 h in the absence of light. The water between the mycelia was absorbed using filter paper and weighed to determine the wet weight. The *Aspergillus flavus* mycelia dry weight was determined using a method described previously [31]. The mycelia were filtered through filter paper, collected, placed in a refrigerator at −80 °C for a period of over 8 h, dried using a vacuum freeze dryer for 72 h, and weighed, and the dry weight recorded.

##### Effect of Surfactin on the *F. oxysporum* (Fo17) Mycelia Growth

One liter of Potato Dextrose Broth (PDB) medium was prepared, and surfactin was added to achieve concentrations of 0.00625, 0.0125, 0.025, 0.05, 0.1, 0.25, 0.5, and 1 mg/mL. The control group was not treated with surfactin. The Fo17 strain was cultured in an incubator at 28 °C for 1 d. The OD value was measured to calculate the growth inhibition rate.

##### Scanning Electron Microscopy of *F. oxysporum* (Fo17) Mycelia and Spore Morphology

The mycelia were fixed in 2.5% glutaraldehyde phosphate buffer (pH 7.2) for 16 h in the presence of 0, 0.5, and 1.0 mg/mL surfactin. The samples were rinsed with phosphate buffer 3 times for 15 min each. Samples were dehydrated using ethanol gradients of 30%, 50%, 70%, 90%, 95%, and 100%. The ethanol was then replaced with isoamyl acetate. The samples were placed in a critical point desiccator, exposed to liquid carbon dioxide, and heated to a temperature above the critical point (31.4 °C, 7.38 × 10^6^ Pa) to vaporize. The dried samples were pasted onto the samples with an electroscopic conductive adhesive. Samples were placed in a vacuum coater for coating, observed under a scanning electron microscope (Olympus BX43 Microscope, Olympus Corporation, Shinjuku-ku, Tokyo, Japan), and photographed.

##### Effect of Surfactin on the Sporulation of *F. oxysporum* (Fo17)

One liter of carboxymethylcellulose medium was prepared, and surfactin was added to achieve concentrations of 0.00625, 0.0125, 0.025, 0.05, 0.1, 0.25, 0.5, and 1 mg/mL. The control group was not exposed to surfactin. Fungal patties with diameters of 4 mm were produced using a hole punch on a spore-laden solid medium, then subsequently inoculated in carboxymethylcellulose sodium medium (CMC) and incubated at 28 °C for 7 d. The fungal suspension was vortexed using a multi-tube vortex mixer at 2500 rpm for 10 min and filtered through four layers of gauze. A volume of 10 μL was then transferred to a hematocrit plate counter, and the number of spores was counted using a microscope (CKX41 inverted microscope, Olympus Corporation, Shinjuku-ku, Tokyo, Japan).

#### 4.2.2. Effect of Surfactin on the Release of Nucleic Acids and Proteins from *F. oxysporum* (Fo17)

The effect of surfactin on the leakage from *F. oxysporum* cells was quantified according to the method of Shao et al. [32]. A spore suspension (1 × 10^6^ spores/mL) was added to 100 mL of Potato Dextrose Broth (PDB) medium, and the mixture was incubated for 3 d at 28 °C. Subsequently, cells were rinsed twice with 50 mM PBS (pH 7.2).

Following centrifugation at 8000 rpm and 4 °C for 5 min, 3 g of mycelia was resuspended in sterile distilled water to 0.00625, 0.0125, 0.025, 0.05, 0.1, 0.25, and 1 mg/mL surfactin concentrations and incubated at 28 °C for 3 h. The nucleic acid and protein concentrations were determined by measuring the absorbance of the supernatant at 260 and 280 nm wavelengths, respectively [33].

#### 4.2.3. Effect of Surfactin on Intracellular ROS Levels and *F. oxysporum* (Fo17) Production Pathways

The ROS inhibitors (tacrolimus (FK506), a calcium-modulated phosphokinase-specific inhibitor; N-acetylcysteine (NAC); and dimethyliodophenylphenon (DPI)) were added to the Fo17 culture medium containing surfactin. Subsequently, the organisms were collected in a freeze-dryer and weighed. Samples were prepared according to the DCFH-DA (2,7-Dichlorofluorescein diacetate) Fluorescent Probe Kit instructions. The fluorescence intensity was observed under a laser scanning copolymer microscope (FV1000, Olympus Corporation, Shinjuku-ku, Tokyo, Japan) at an excitation wavelength of 488 nm, and the fluorescence intensity was measured using an enzyme-labeled instrument (Varioskan LUX, Thermo Fisher Scientific, Waltham, MA, USA). Relative fluorescence intensity per unit mass of *F. oxysporum* was calculated. In addition, the amount of T-2 toxin synthesized was examined.

#### 4.2.4. *Yap1* Mediates a Key Role in *Tri5* Response to Surfactin Stress

The aim of this study was to investigate the effects of surfactin on the growth and virulence of *F. oxysporum* in the absence of *Yap1*. This was achieved by knocking down *Yap1* in *F. oxysporum* using homologous recombination exchange and *Agrobacterium* transformation.

##### Knockout of the *Yap1* Gene in *F. oxysporum*

To ascertain the *Yap1* function in response to surfactin, deletion strains of *Yap1* were generated. Knockout transformants were obtained using the *Yap1* knockout construct with the knockout vector pBluescript KS (±) (pBS)-HPH1. *Yap1* gene sequences were obtained from the *F. oxysporum* gene database (http://www.broadinstitute.org/annotation/genome/fusarium_group/MultiHome.html) (accessed on 6 June 2023), and primers were designed and amplified according to the results of the comparison. The deletion structure was established by double-joint fusion PCR. The purified PCR product was transformed into Fo17 protoplasts. Transformed protoplasts were screened on HYG plates (containing thiamphenicol at 0, 50, and 100 μg/mL concentrations) and incubated at 25 °C for 5–7 days until the transformants grew. Polymerase chain reaction (PCR) was conducted using primers *Yap1*-A1 and *Yap1*-A4, and the results were validated by sequencing analysis.

##### Influence of the *Yap1* Gene on the *F. oxysporum* T-2 Toxin Production

A 1 L volume of Glucose Yeast Malt Agar medium (GYM Agar medium) was prepared, and two 4 mm diameter fungus pies of the wild strain Fo17 and defective strain ∆*Yap1* were inoculated into 10 mL GYM medium. The culture was incubated at 28 °C in alternating light and dark conditions. On day 15, 10 mL of ethyl caproate was added to the culture solution, vortexed at 2500 rpm/min for 20 min, and centrifuged at 10,000 rpm for 10 min. The extraction was repeated thrice, and the upper layer of the solution was collected and dried in nitrogen. The precipitate was shaken with 1 mL of 30% aqueous methanol, filtered through a 0.22 μm microporous filter membrane, and analyzed for T-2 toxin.

##### GFP Green Fluorescent Labeling of the *Yap1* Gene

The genomic DNA of *F. oxysporum* protoplasts was used as a template, and the sequences were amplified using the corresponding primers. PCR products were purified and digested. The digested products were ligated into pCAMBIA1300-neo (kanamycin) and transformed into *Escherichia coli* receptor DH-5α. The plasmids were extracted for use in subsequent experiments.

Construction of the knockout vector:

Take 200 µL of *Agrobacterium* susceptible cells and melt them at 4 °C, then add 1 µg of plasmid NDA and mix well, and leave for 0.5 h. Add liquid nitrogen and freeze for 60 s, then react at 37 °C for 5 min. Inoculate the mixtures into Luria–Bertani (LB) selection medium. Plasmid DNA from the monoclonal colonies was extracted and identified using PCR.

*Agrobacterium*-mediated transformation of *F. oxysporum*:

The *F. oxysporum Yap1* knockout strain was harvested and added to 100 mL of sodium carboxymethyl cellulose spore culture medium and incubated at 25 °C and 160 rpm for 5–7 days. The culture medium was filtered through gauze to collect the filtrate, and the precipitate was centrifuged at 4 °C for 10 min at 2500 rpm. The spores were adjusted to a concentration of 1 × 10^7^ cfu/mL. The *Agrobacterium* monoclonalis was inoculated onto the Lysogenic Broth medium (LB) plates (containing rifampicin 50 μg/mL, kanamycin 50 μg/mL) and incubated at 28 °C for 4–5 days to obtain the monoclonal organisms. Subsequently, the *Agrobacterium* was inoculated into 100 mL of LB liquid medium (containing rifampicin 50 μg/mL, kanamycin 50 μg/mL) and incubated at 28 °C with agitation at 250 rpm for 1–2 days. From this culture, 10 mL of *Agrobacterium* were taken. The culture medium was centrifuged at 24 °C and 4000 rpm for 10 min. The resulting precipitate was then transferred to 0.5 mL IM liquid medium and mixed thoroughly. This mixture was then centrifuged at 24 °C and 4000 rpm for 10 min. Afterwards, the sample was centrifuged for 10 min at 24 °C and 4000 rpm. Subsequently, the resulting precipitate was then transferred to 5 mL IM liquid medium (containing 200 μM acetosyringone), and the sample was incubated at 100 rpm for 4–5 h at 28 °C until the OD (Optical Density) 600 of the bacterial liquid reached 0.8. A 0.45 μM nitrocellulose membrane was placed on the surface of the IM plate (containing 200 μM acetosyringone). The aforementioned *Agrobacterium* liquid was pipetted along with the fungal spore solution, with a concentration of 1 × 10^7^ cfu/mL. The mixture was evenly coated on the nitrocellulose membrane and placed in an incubator at 25 °C for 3–5 days. These samples were then transferred to PDA (Potato Dextrose Agar) medium at 28 °C until the bacterial body grew. Thereafter, the bacterial transformant was transferred to the screening medium for culture, with the objective of purifying the strains and carrying out single-spore cultivation. The genomic DNA of the strain to be validated was extracted and amplified by PCR with the primers neo-F and neo-R, backfill-F and backfill-R, and GFP-F and GFP-R. Validation of backfilled strains was performed.

##### Subcellular Localization of the *Yap1* Gene in *F. oxysporum* Subjected to Surfactin Stress

The subcellular distribution of *Yap1* subjected to surfactin stress was determined using DAPI dye and fluorescence microscopy. The *Yap1*-GFP strain was treated with 0, 0.5 MIC, and 1 MIC surfactin, then incubated with 1 mg/mL DAPI staining solution for 10 min. Subsequently, cover slips were added to the samples to create temporary slides for observation of subcellular localization under a fluorescence microscope (Olympus BX43 Microscope, Olympus Corporation, Japan).

### 4.3. Data Processing

The data were processed using GraphPad Prism 9.0 with SPSS 22.0. The results are presented as mean ± standard deviation. The significant level for the statistical tests was set at *p* < 0.05.

## 5. Conclusions

Mycelial growth and T-2 toxin biosynthesis were significantly inhibited in *F. oxysporum* following exposure to surfactin. The accumulation of intracellular ROS triggered the entry of the ROS transcription factor *Yap1* into the nucleus, resulting in the inhibition of *Tri5* expression and the subsequent blockage of T-2 toxin synthesis. Thus, surfactin acted as a fungal inhibitor. This study provides a theoretical foundation for the prevention and control of *F. oxysporum* and associated toxin contamination in dried fish. Surfactin could, therefore, be used in the prevention and control of fungi and associated toxins in the dried aquatic products industry.

## Data Availability

Data is contained within the article and Supplementary Material.

## Supplementary Materials

The following supporting information can be downloaded at: https://www.mdpi.com/article/10.3390/molecules29225402/s1: Table S1. Amplification primers, Table S2. Transposon validation primers, Table S3. Primers, GenBank accession numbers and amplification procedures used for strain identification, Figure S1. Neighbor-joining tree constructed based on the internal transcribed spacer (OP412776, A), translation elongation factor 1-alpha (OP330068, B) and β-tubulin (OP382711, C) sequences. Total ions chromatogram for T-2 toxin produced by Fusarium oxysporum (Fo17, GDMCC60824) (D). Appendixes SA–SE. Sequencing results. Reference [34] is cited in the Supplementary Materials.
