# Supplementary material for: Molecular Mechanism: Inhibition of Fusarium oxysporum T-2 Toxin Synthesis by Surfactin in Dried Fish: Induction of Yap1 Nucleation by ROS Accumulation
Source: Molecules. 2024 Nov 15;29(22):5402. doi: 10.3390/molecules29225402 (PMC11597049; doi:10.3390/molecules29225402)
Supplement: Supplementary file 1 [file molecules-29-05402-s001.zip › molecules-3252030-supplementary.pdf]

Appendix SA：Primer names and sequences

Table S1. Amplification primers

Primer name	Primer sequence（5′ to 3′）
*Yap1*_5F	CACAGGCTTCAGGCACAGACAGGCTC
*Yap1*_3R	TTGGGCAACACAACTACTGGAGACGTC
*Yap1*_split_A1	CCTGCTCTTCGCTGCTCTGAACTC
*Yap1*_split_A2	GTCGTGACTGGGAAAACCCTGGCGGCCTTCTCT AGCTCTTCAACCT
*Yap1*_split_A3	TCCTGTGTGAAATTGTTATCCGCTCTACTTTGAC TGGCTGTCACAACAG
*Yap1*_split_A4	CTTAATCATTGTGTAGCTGTGTGCTGG
Yap1-A1	TGTCTAGACGATGGCTTCTACTGGTACTGGAGG
Yap1-A2	ATGGATCCGCCTTCTCTAGCTCTTCAACCTTTGT CTCAAG
Yap1-A3	ATCTCGAGGCACCAACTTTGACTGGCTGTCACA AC
Yap1-A4	ATGGTACCTTGGGCAACACAACTACTGGAGACG TC
Yap1-A5	TCAGCGTGCTTTCAGAGAACGCAAGG
Yap1-A6	CAAGAGCATCGTTGAAGAAGTCGTCG
hygromycin_5F	ATGAAAAAGCCTGAACTCACCGCGAC
hygromycin_3R	CTATTCCTTTGCCCTCGGACGAGTGCTG
M13-F	CGCCAGGGTTTTCCCAGTCACGAC
M13-R	AGCGGATAACAATTTCACACAGGA
HY	GGATGCCTCCGCTCGAAGTA
YG	CGTTGCAAGACCTGCCTGAA
5UTR-F-KpnI	CGGGGTACCAAGTACTTTCGCATCCAGTG
3UTR-R-SalI	ACGCGTCGACAGTAGGACTTGTTCAAGATGGT
5UTR-F-KpnI	CGGGGTACCAAGTACTTTCGCATCCAGTG
Yap1-R-BamHI	CGCGGATCCCCCACCTCCAATCGACAGACCAAG ATGCT
GFP-F-BamHI	CGCGGATCCGGCGGTGGTGGGAGTATGGTGAGC AAGGGCGAGGA
GFP-R	CTTTGTTCATGAACCTGGTCTCACTTGTACAGCT CGTCCATGC
3UTR-F	GCATGGACGAGCTGTACAAGTGAGACCAGGTTC ATGAACAAAG
3UTR-R-SalI	ACGCGTCGACAGTAGGACTTGTTCAAGATGGT
GFP-F-BamHI	CGCGGATCCGGCGGTGGTGGGAGTATGGTGAGC AAGGGCGAGGA
3UTR-R-SalI	ACGCGTCGACAGTAGGACTTGTTCAAGATGGT

Table S2. Transposon validation primers

Primer name	Primer sequence（5′ to 3′）
backward complementary verification-F	GTGACCCATGGCGATGCCTGCTTGC
backward complementary verification -R	TCAACTGACAAGCCTCATGAGCTC
NEO-F	GATTGAACAAGATGGATTGCACGCAGG
NEO-R	GGTACCTCAGAAGAACTCGTCAAGAAG
GFP-F-BamHI	CGCGGATCCGGCGGTGGTGGGAGTATGGTG AGCAAGGGCGAGGA
GFP-R	CTTTGTTCATGAACCTGGTCTCACTTGTACA GCTCGTCCATGC
GFP-F1-3567	CGTGACCACCCTGACCTA
GFP-R1-3869	ATGCCGTTCTTCTGCTTG

Appendix SB: Nucleotide sequence of the coding region of the *Yap1* gene

1.*Yap1* 5'.seq

TTCGCCTGCTCCGTGACCTGGACTGGCAGAGAGCTTGAACTATAATCAAACCGCGCG
GCATCAGTTTCGTCTGCCTCGATTTCAAATCGTTCTTCACCGTTACGCCTCTTTTCGATC
GCCCGTTTCATATTTCGCGATGACTTTGCCACCCAACTTCCTCTTGACCCCGCAACAG
CAGAACCTGCTCTTCGCTGCTCTGAACTCCAACAAGCAGCAGCTCTCTGGTTCTTCCG
TGGACAATTCTCTGACGGTTTCTCCCACGACGTCCCGCAATTCTGCTGGCCAACAAAA
GCCCGCCACCGCTACAGGTTACCAGGAGAGTCCCTTCCTCGACAACTACGACTATGA
CTTTGGCGATTCTGGTTTCGACTTCTCCTTCGCCAGCGAGGACCAGCCTTCCATGATTG
GAGATCTTCCTGCTGCTTCCACGGAGCCCAACAACAACTCTGGTTCTGTCGCTCTGTC
CGACTCCCCAGAGACTGATACTCCAGAGAAGCGCAGCTACCCCGACGACGAGGATG
ACGAAGATAGCCCTGGTGGGGATCACAAGCGCCGTGAGAGTACCGACAAGGTCCCT
AAGAAGCCCGGTCGTAAGCCTCTTACATCCGAGCCCAGCTCGGTACGTTTTATCAATT
GATTACATATAAAATTTACTTACTAATCCAAACAATAGAAGCGCAAGGCTCAAAATC
GAGCCGCTCAGCGTGCTTTCAGAGAACGCAAGGAGAAGCATTTGAAGGACCTTGAG
ACAAAGGTTGAAGAGCTAGAGAAGGCTTCGCAGGCTGCCAACCATGAGAATGGCAT
GCTTCGAGCTCAGGTTGAGCGCATGACAGCTGAACTCAACCAGTATAAGCAGAAGGT
CACAGTGATGTCAGCTACCAAGTCACTGCCCAGAGAAAAGGTTCCTTTCGGCAGCGC
TGCCGTCAGCAACCTCGGCGATGTTACTTCCAGTTCGAGTTTCCAAATTTGGCATGCTT
CCAGGACC

2. Sequencing splicing results of HYG gene amplification products (*Yap1* knockout validation)

GCCTGAACTCACCGCGACGTCTGTCGAGAAGTTTCTGATCGAAAAGTTCGACAGCGT
CTCCGACCTGATGCAGCTCTCGGAGGGCGAAGAATCTCGTGCTTTCAGCTTCGATGTA
GGAGGGCGTGGATATGTCCTGCGGGTAAATAGCTGCGCCGATGGTTTCTACAAAGAT
CGTTATGTTTATCGGCACTTTGCATCGGCCGCGCTCCCGATTCCGGAAGTGCTTGACA
TTGGGGAGTTCAGCGAGAGCCTGACCTATTGCATCTCCCGCCGTGCACAGGGTGTCA
CGTTGCAAGACCTGCCTGAAACCGAACTGCCCGCTGTTCTCCAGCCGGTCGCGGAGG
CCATGGATGCGATCGCTGCGGCCGATCTTAGCCAGACGAGCGGGTTCGGCCCATTCG
GACCGCAAGGAATCGGTCAATACACTACATGGCGTGATTTCATATGCGCGATTGCTG

ATCCCCATGTGTATCACTGGCAAACTGTGATGGACGACACCGTCAGTGCGTCCGTCGC
GCAGGCTCTCGATGAGCTGATGCTTTGGGCCGAGGACTGCCCCGAAGTCCGGCACCT
CGTGCATGCGGATTTCGGCTCCAACAATGTCCTGACGGACAATGGCCGCATAACAGC
GGTCATTGACTGGAGCGGGGCGATGTTCGGGGATTCCCAATACGAGGTCGCCAACAT
CCTCTTCTGGAGGCCGTGGTTGGCTTGTATGGAGCAGCAGACGCGCTACTTCGAGCGG
AGGCATCCGGAGCTTGCAGGATCGCCGCGCCTCCGGGCGTATATGCTCCGCATTGGT
CTTGACCAACTCTATCAGAGCTTGGTTGACGGCAATTTCGATGATGCAGCTTGGGCGC
AGGGTCGATGCGACGCAATCGTCCGATCCGGAGCCGGGACTGTCGGGCGTACACAA
ATCGCCCGCAGAAGCGCGGCCGTCTGGACCGATGGCTGTGTAGAAGTACTCGCCGAT
AGTGGAAACCGACGCCCCAGCACTCGTCCGAGG

3. *Yap1*-5'-HY sequencing results (*Yap1* knockout validation)
GGAGAGCTCTCTGGTTCTTCCGTGGACAATTCTCTGACGGTTTCTCCCACGACGTCCC
GCAATTCTGCTGGCCAACAAAGCCCGCCACCGCTACAGGTTACCAGGAGAGTCCCT
TCCTCGACAACTACGACTATGACTTTGGCGATTCTGGTTTCGACTTCTCCTTCGCCAGC
GAGGACCAGCCTTCCATGATTGGAGATCTTCCTGCTGCTTCCACGGAGCCCAACAAC
AACTCTGGTTCTGTCGCTCTGTCCGACTCCCCAGAGACTGATACTCCAGAGAAGCGCA
GCTACCCCGACGACGAGGATGACGAAGATAGCCCTGGTGGGGATCACAAGCGCCGT
GAGAGTACCGACAAGGTCCCTAAGAAGCCCGGTCGTAAGCCTCTTACATCCGAGCCC
AGCTCGGTACGTTTTATCAATTGATTACATATAAAATTTACTTACTAATCCAAACAAT
AGAAGCGCAAGGCTCAAAATCGAGCCGCTCAGCGTGCTTTCAGAGAACGCAAGGAG
AAGCATTTGAAGGACCTTGAGACAAAGGTTGAAGAGCTAGAGAAGGCGGATCCTGG
AGGTCAACACTGAATGCCTATTTTGGTTTAGTCGTCCAGGCGGTGAGCACAAAATTTG
TGTCGTTTGACAAGATGGTTCATTTAGGCAACTGGTCAGATCAGCCCCACTTGTAGCA
GTAGCGGCGGCGCTCGAAGTGTGACTCTTATTAGCAGACAGGAACGAGGACATTATT
ATCATCTGC.TGCTTGGTGCACGATAACTT..GGTGCGTTTGTCAAGCAAGG.TAAGTGG
ACGACCCGGT.CATACCTTCTTAAGTTCGCCCTTCCTCCC.TTTATTTCAGATTCAATCTG
ACTTACCTATTCTACCC.AAGCATCCAAATGAAAAAGCCTGAACTCACCGCGACGTCT
GTCGAGAAGTTTCTGATCGAAAAGTTCGACAGCGTCTCCGACCTGATGCAGCTCTCG
GAGGGCGAAGAATCTCGTGCTTTCAGCTTCGATGTAGGAGGGCGTGGATATGTCCTG
CGGGTAAATAGCTGCGCCGATGGTTTCTACAAAGATCGTTATGTTTATCGGCACTTTG
CATCGGCCGCGCTCCCGATTCCGGAAGTGCTTGACATTGGGGAGTTCAGCGAGAGCC
TGACCTATTGCATCTCCCGCCGTGCACAGGGTGTCACGTTGCAAGACCTGCCTGAAAC
CGAACTGCCCGCTGTTCTCCAGCCGGTCGCGGAGGCCATGGATGCGATCGCTGCGGC
CGATCTTAGCCAGACGAGCGGGTTCGGCCCATTCGGACCGCAAGGAATCGGTCAATA
CACTACATGGCGTGATTTCATATGCGCGATTGCTGATCCCCATGTGTATCACTGGCAA
ACTGTGATGGACGACACCGTCAGTGCGTCCGTCGCGCAGGCTCTCGATGAGCTGATG
CTTTGGGCCGAGGACTGCCCCGAAGTCCGGCACCTCGTGCATGCGGATTTCGGCTCCA
ACAATGTCCTGACGGACAATGGCCGCATAACAGCGGTCATTGACTGGAGCGGGGCG
ATGTTCGGGGATTCCCAATACGAGGTCGCCAACATCCTCTTCTGGAGGCCGTGGTGG..
CTGTGAGCAGCCCCC

4. Yap1-3'-YG sequencing results (Yap1 knockout validation)
CAGCCGGTCGCGGAGGCCATGGATGCGATCGCTGCGGCCGATCTTAGCCAGACGAGC
GGGTTCGGCCCATTCGGACCGCAAGGAATCGGTCAATACACTACATGGCGTGATTTC
ATATGCGCGATTGCTGATCCCCATGTGTATCACTGGCAAACTGTGATGGACGACACC
GTCAGTGCGTCCGTCGCGCAGGCTCTCGATGAGCTGATGCTTTGGGCCGAGGACTGCC
CCGAAGTCCGGCACCTCGTGCATGCGGATTTCGGCTCCAACAATGTCCTGACGGACA
ATGGCCGCATAACAGCGGTCATTGACTGGAGCGGGGCGATGTTCGGGGATTCCCAAT
ACGAGGTCGCCAACATCCTCTTCTGGAGGCCGTGGTTGGCTTGTATGGAGCAGCAGA
CGCGCTACTTCGAGCGGAGGCATCCGGAGCTTGCAGGATCGCCGCGCCTCCGGGCGT
ATATGCTCCGCATTGGTCTTGACCAACTCTATCAGAGCTTGGTTGACGGCAATTTCGA
TGATGCAGCTTGGGCGCAGGGTCGATGCGACGCAATCGTCCGATCCGGAGCCGGGAC
TGTCGGGCGTACACAAATCGCCCGCAGAAGCGCGGCCGTCTGGACCGATGGCTGTGT
AGAAGTACTCGCCGATAGTGGAAACCGACGCCCCAGCACTCGTCCGAGGGCAAAGG
AATAGAGTAGGTCGACCTCGAGGCACCAACTTTGACTGGCTGTCACAACAG..AACGG
TGGACAGTTTGACCCACAGCTCTTTGGTGATTATCG.TGAGCCTCAAGAAAACGTATTG
GCAAATC.CATCTTTCGACGACTTCTTCAACGATGCTCTTGACAGCGATTTCTTTACTCC
TTACAACATGGCTC..CTAACAGTCCCAGCGCGCATCTCAATGGCCAGGCCAAGAAGC
CATCGAACCTGATTGATCAGATTGATGCTCAAAAGGAGTCCGATGACGAACCTCTCA
AGAAGCAGAACATGAACTGTAATCAACTGTGGTATGTATCAACTTATAGAATCAGGA
AATGTCTGTCTCTAACACGTTTCTACAGGGAGAAACTTCAAGCTTGCCCCAAGGCAC
AGAATGGTGAATTCGACCTCGACGGCCTCTGCTCTGAACTTACCAAGAAGGCCAAGT
GCTCTGGCACTGGTCCTGTGGTTGCTGAGACCGACTTTGCGATGCATTCTGCGCAACG
CATACGTACGTAACCA

5. *Yap1*-5'-HY and *Yap1*-3'-YG splice sequences
GGAGAGCTCTCTGGTTCTTCCGTGGACAATTCTCTGACGGTTTCTCCCACGACGTCCC
GCAATTCTGCTGGCCAACAAAAGCCCGCCACCGCTACAGGTTACCAGGAGAGTCCCT
TCCTCGACAACTACGACTATGACTTTGGCGATTCTGGTTTCGACTTCTCCTTCGCCAGC
GA..GGACCAGCCTTC..CATGATTGGAGATCTTCCTGCTGCTTCCACGGAGCCCAACAA
CAACTCTGGTTCTGTCGCTCTGTCCGACTCCCCAGAGACTGATACTCCAGAGAAGCGC
AGCTACCCCGACGACGAGGATGACGAAGATAGCCCTGGTGGGGATCACAAGCGCCG
TGAGAGTACCGACAAGGTCCCTAAGAAGCCCGGTCGTAAGCCTCTTACATCCGA.GCC
CAGCTCGGTACGTTTTATCAATTGATTACATATAAAATTTACTTACTAATCCAAACAA
TAGAAGCGCAAGGCTCAAAATCGAGCCGCTCAGCGTGCTTTCAGAGAACGCAAGGA
GAAGCATTTGAAGGACCTTGAGACAAAGGTTGAAGAGCTAGAGAAGGCGGATCCTG
GAGGTCAACACTGAATGCCTATTTTGGTTTAGTCGTCCAGGCGGTGAGCACAAAATTT
GTGTCGTTTGACAAGATGGTTCATTTAGGCAACTGGTCAGATCAGCCCCACTTGTAGC
AGTAGCGGCGGCGCTCGAAGTGTGACTCTTATTAGCAGACAGGAACGAGGACATTAT
TATCATCTGCTGCTTGGTGCACGATAACTTGGTGCGTTTGTCAAGCAAGGTAAGTGGA
CGACCCGGTCATACCTTCTTAAGTTCGCCCTTCCTCCCTTTATTTCAGATTCAATCTGA
CTTACCTATTCTACCCAAGCATCCAAATGAAAAAGCCTGAACTCACCGCGACGTCTG
TCGAGAAGTTTCTGATCGAAAAGTTCGACAGCGTCTCCGACCTGATGCAGCTCTCGG
AGGGCGAAGAATCTCGTGCTTTCAGCTTCGATGTAGGAGGGCGTGGATATGTCCTGC
GGGTAAATAGCTGCGCCGATGGTTTCTACAAAGATCGTTATGTTTATCGGCACTTTGC

ATCGGCCGCGCTCCCGATTCCGGAAGTGCTTGACATTGGGGAGTTCAGCGAGAGCCT
GACCTATTGCATCTCCCGCCGTGCACAGGGTGTCACGTTGCAAGACCTGCCTGAAAC
CGAACTGCCCGCTGTTCTCCAGCCGGTCGCGGAGGCCATGGATGCGATCGCTGCGGC
CGATCTTAGCCAGACGAGCGGGTTCGGCCCATTCGGACCGCAAGGAATCGGTCAATA
CACTACATGGCGTGATTTCATATGCGCGATTGCTGATCCCCATGTGTATCACTGGCAA
ACTGTGATGGACGACACCGTCAGTGCGTCCGTCGCGCAGGCTCTCGATGAGCTGATG
CTTTGGGCCGAGGACTGCCCCGAAGTCCGGCACCTCGTGCATGCGGATTTCGGCTCCA
ACAATGTCCTGACGGACAATGGCCGCATAACAGCGGTCATTGACTGGAGCGGGGCG
ATGTTCGGGGATTCCCAATACGAGGTCGCCAACATCCTCTTCTGGAGGCCGTGGTTGG
CTTGTATGGAGCAGCAGACGCGCTACTTCGAGCGGAGGCATCCGGAGCTTGCAGGAT
CGCCGCGCCTCCGGGCGTATATGCTCCGCATTGGTCTTGACCAACTCTATCAGAGCTT
GGTTGACGGCAATTTCGATGATGCAGCTTGGGCGCAGGGTCGATGCGACGCAATCGT
CCGATCCGGAGCCGGGACTGTCGGGCGTACACAAATCGCCCGCAGAAGCGCGGCCG
TCTGGACCGATGGCTGTGTAGAAGTACTCGCCGATAGTGGAAACCGACGCCCCAGCA
CTCGTCCGAGGGCAAAGGAATAGAGTAGGTCGACCTCGAGGCACCAACTTTGACTGG
CTGTCACAACAG..AACGGTGGACAGTTTGACCCACAGCTCTTTGGTGATTATCGTGAG
CCTCAAGAAAACGTATTGGCAAATCCATCTTTCGACGACTTCTTCAACGATGCTCTTG
ACAGCGATTTCTTTACTCCTTACAACATGGCTCCTAACAGTCCCAGCGCGCATCTCAA
TGGCCAGGCCAAGAAGCCATCGAACCTGATTGATCAGATTGATGCTCAAAAGGAGTC
CGATGACGAACCTCTCAAGAAGCAGAACATGAACTGTAATCAACTGTGGTATGTATC
AACTTATAGAATCAGGAAATGTCTGTCTCTAACACGTTTCTACAGGGAGAAACTTCA
AGCTTGCCCCAAGGCACAGAATGGTGAATTCGACCTCGACGGCCTCTGCTCTGAACTT
ACCAAGAAGGCCAAGTGCTCTGGCACTGGTCCTGTGGTTGCTGAGACCGACTTTGCG
ATGCATTCTGCGCAACGCATACGTACGTAACCA

Appendix SC: Sequencing results of genes

1. 5UTR-*Yap1*-3UTR

AAGTACTTTCGCATCCAGTGAGCTGTTTTCTGGCAGCCGTTGAGATACGAAATTACGG
TTGAAATTTCAGCGATTGATAACTGGACTTGACCCATTGGGAAGAATAATTGCCATTG
ATTGGTCGAAAGCGAGGTGAATGCGCCGTGTGGCAAACTCCGAATCTTGGTCTAAAT
CCGGGGGCGGGGAGAACTTCGACAGGACCGCGTGGTTGTGGCTCGGGGATAAGGCA
ATGGCTGCAGATGGGGTGAAACGGGGCGTTGTTAGCAGTCAGAAACAGTTAGCTATG
AGCTCATGAGGCTTGTCAGTTGAAGCATAGCCAAGGTCATGGTCTCGAGTCAATTCTG
CATTGCAGTTGAGACAGAAGCCCAAGGCCGGCCATTACAGCAAGTAAACGACATTCT
GTCGCAGTCGGAGAAGCATAATGCCGTTCAGGAGGAATCGCCACTGCCAAACAAGA
CAAGGTCGAGAAAGGGGGAGAAAGCTCGAGAGACTCGTGCACAACGTCGAAGGGC
GTGCATGTCAACTAATAGTATCTGCCGAAAACGATCGGGTATAAGTCGGTCACAGGC
AACGTGCCAATAATTGAAGGCTCATGGCCAAACGTCTTTTATTGTGAGTCAGAGCCTC
AGACTCTAGAGAAGAGAGAAAAGACAGACAATGACATGCTCATGGCTCGTTTTGGC
ATGGCCTAGATGGGCTTTTTGTTTCGTCCTCTCTGTTGTAAGATATCCATGGGTCCCGA
GGACCGGTAGCCCCGGCTGACAAATGCAACTTTGACGCTATTCGCACTGTCGAGAGT
TCACGTTCCTGTACCCAGCCTCTCTCGGAAACTCGCTGACCCCCACCCAGAATGGTTC
CTGTTCAGGGTCTAGTGAGCTGATGGAAGAATGAACTAGAAGGAATCTTTGTGACTC
AGGCAGCAAATTTTCTAGTCCGACGCTCCGCATTGGACCCTTGCCAGCTTGCCTTTGC
TTGCTCTGGTACGCGTAGATACAGTACCTACAGTGCGATTCAACGCCCGCTCAGCTGC

CCCCGCTCTACCTACTGCAGCCATCCATCTCACTCGATTTCCCTTCCTCGCCCATTCTT
GGCTCCGCCTGACACCAGAAACCACCGATTTCATCATCAATCTTACATACTGTCACCT
TACATACCATAGTAGCTTCAGTTTCTATCCTGTCCTTTGCGGCCTGAATCCGAAAACTC
CGTTCTTCTTATTTTCAACTTCGCCTCTCTTCTCATCGCCTTTTGACAATTATATGCCAG
TATCATAACCCAGCAGAAGCCCGCCCCGCTCGTCAAGGTCCCCGACGTCGCCGCTAC
ACAGGCTTCAGGTACAGACAGGCTCTGGTCTGGCTCTTTGTCCCCTCCTGCGTGAGCC
TGGACTGGCAGAGAGCTTGAACCTATAATCAAACCGCGCGGCATCAGTTTCGTCTGC
CTCGATTTCAAATCGTTCTTCACCGTTACGCCTCTTTTCGATCGCCCGTTTCATATTTCG
CGATGACTTTGCCACCCAACTTCCTCTTGACCCCGCAACAGCAGAACCTGCTCTTCGC
TGCTCTCAACTCCAACAAGCAGCAGCTCTCTGGTTCTTCCGTGGACAATTCTCTGACG
GTTTCTCCCACGACGTCCCGCAATTCTGCTGGCCAACAAAAGCCCACCACCGCTACA
GGTTACCAGGAGAGTCCCTTCCTCGACAACTACGACTATGACTTTGGCGATTCTGGTT
TCGACTTCTCCTTCGCCAGCGAGGACCAGCCTTCCATGATTGGAGATCTTCCTGCTGCT
TCCACGGAACCCAACAACAACTCTGGTTCTGTCGCTCTGTCCGACTCCCCAGAGACTG
ATACTCCAGAGAAGCGCAGCTACCCCGACGACGAGGATGACGAAGATAGCCCTGGT
GGGGATCACAAGCGCCGTGAGAGTACCGACAAGGTCCCTAAGAAGCCCGGTCGTAA
GCCTCTTACATCCGAGCCCAGCTCGGTACGTTTTATCAATTGATTACATATAAAATTTA
CTTACTAATCCAAACAATAGAAGCGCAAGGCTCAAAATCGAGCCGCTCAGCGTGCTT
TCAGAGAACGCAAGGAGAAGCATTTGAAGGACCTTGAGACAAAGGTTGAAGAGCTA
GAGAAGGCTTCGCAGGCTGCCAACCATGAGAATGGCATGCTTCGAGCTCAGGTTGAG
CGCATGACAGCTGAACTCAACCAGTATAAGCAGAAGGTCACAGTGATGTCAGCTACC
AAGTCACTGCCCAGAGAAAAGGTTCCTTTCGGCAGCGCTGCCGTCAGCAACCTCGGC
GATGTTAACTTCCAGTTCGAGTTTCCCAAATTTGGCATGCTTCCAGGACCACCTGTCA
GCAAGACTGGCTCATCGCCTACAAGCCCGGATCAGCAAAAGATCACATACCCAAGC
CCTACCAACAGCCTCAACAACAGCGCGCAATCCGCACAACAATTCAAGGATGACTTG
GCCAAGTTCTCAGGTGTCTTCAGCCCTTCCATGGCAAGCTCTGCCACCAACCCCTCTC
GTGCGAGTGTTGACTCTGCAAACTACAGCGTCAATGGCGCTTCCAGCTCCCCATCTGC
TTCATCTCATTCAAACACTGGTCCCAGCTCTTCTTGCGGAACATCGCCTGAGCCTTTCA
ACCAGTCTCCCATGGGCTTCAAGCCCGTTGATACGATGACTACCATTGGCGAGGAGC
AGACATATCAGAACAGCAACAACAACCCCAGTCAATTCGGCAATATTGATCTCAACA
GCACCAACTTTGACTGGCTGTCACAACAGAACGGTGGACAGTTTGACCCACAGCTCT
TTGGTGATTATCGTGAGCCTCAAGAAAACGTATTGGCAAATCCATCTTTCGACGACTT
CTTCAACGATGCTCTTGACAGCGATTTCTTTACTCCTTACAACATGGCTCCTAACAGTC
CCAGCGCGCATCTCAATGGCCAGGCCAAGAAGCCATCGAACCTGATTGATCAGATTG
ATGCTCAAAAGGAGTCCGATGACGAACCTCTCAAGAAGCAGAACATGAACTGTAAT
CAACTGTGGTATGTATCAACTTATAGAATCAGGAAATGTCTGTCTCTAACACGTTTCT
ACAGGGAGAAACTTCAAGCTTGCCCCAAGGCACAGAATGGTGAATTCGACCTCGAC
GGCCTCTGCTCTGAACTTACCAAGAAGGCCAAGTGCTCTGGCACTGGTCCTGTGGTTG
CTGAGACCGACTTTGACACCATTCTGCAAAAGTATATGGGCAAAGACGTCTCCAGTA
GTTGTGTTGCCCAACAGTTGGGTGTGGAAATAAAGTCGAGTGAACCAAAGCAAGATA
AGCATCTTGGTCTGTCGATTTGAGACCAGGTTCATGAACAAAGATTCGGCTGTACACT
ACAAACTTCGGCGGCATACCCAGCACACAGCTACACAATGATTAAGGCGTTTACAAC
AGACAGGATGGGATCGGAAAACCAAGGGATTCCAGCGCTTGATTTACCTCCGTCAAC
GGAATTGGATGCATGGAAAGGAGAAAGGATGATCTATGGTATGATTATATAACATGC

CATGAAATCTTCGACCTGTTAACTCAGCTTGAAATTTCTTTGGGGATTTACTTGTGCTT
TTTTTTTGGATGGACATTTTTTTGCTGCTATGAATAATTATGACTTATTTCTAGTTTCAA
TTGTCAAGGGCTCAACAGGGGATATATGATGATCACTGTGACTTGGCAACGCACATG
TTGATCAGATATGTACATCAAAGGGACTATTCAACTTTCATTACTACGTAAAGATGAT
CAAAGTGTTGGAGGCGCTCTTGTGATACGAGTATTAGTATCCAAGGATATAAATTGC
ACGCTACAGCAGCAACTTTGAGCCAATCTGCAACATCCAGACCCTGCATACGTCAGT
AAATTAAGATCTAAAAAAGGTTCAAACGCAAGTAGAAGATCGCCGTGACATCATTTT
TGGGTCTATCATTGACTGAAAGCTGTTGTGATTTTTTATCATGACATCTAATCTTAGTC
ATCAAGTGGAGCCAGATGATCGCGACAAGGAAGCTTTAGCCAGACCAAACCATCTTG
AACAAGTCCTACTCAGCTTACAA

5UTR-F-KpnI： CGGGGTACCAAGTACTTTCGCATCCAGTG

3UTR-R-SalI： ACGCGTCGACAGTAGGACTTGTTCAAGATGGT

Yap1 sequence in yellow font, 5UTR and 3UTR in blue font at both ends, and termination codon in red font.

2. 5UTR-*Yap1*-2GGGGS-GFP-3UTR

AAGTACTTTCGCATCCAGTGAGCTGTTTTCTGGCAGCCGTTGAGATACGAAATTACGG
TTGAAATTTCAGCGATTGATAACTGGACTTGACCCATTGGGAAGAATAATTGCCATTG
ATTGGTCGAAAGCGAGGTGAATGCGCCGTGTGGCAAACTCCGAATCTTGGTCTAAAT
CCGGGGGCGGGGAGAACTTCGACAGGACCGCGTGGTTGTGGCTCGGGGATAAGGCA
ATGGCTGCAGATGGGGTGAAACGGGGCGTTGTTAGCAGTCAGAAACAGTTAGCTATG
AGCTCATGAGGCTTGTCAGTTGAAGCATAGCCAAGGTCATGGTCTCGAGTCAATTCTG
CATTGCAGTTGAGACAGAAGCCCAAGGCCGGCCATTACAGCAAGTAAACGACATTCT
GTCGCAGTCGGAGAAGCATAATGCCGTTCAGGAGGAATCGCCACTGCCAAACAAGA
CAAGGTCGAGAAAGGGGGAGAAAGCTCGAGAGACTCGTGCACAACGTCGAAGGGC
GTGCATGTCAACTAATAGTATCTGCCGAAAACGATCGGGTATAAGTCGGTCACAGGC
AACGTGCCAATAATTGAAGGCTCATGGCCAAACGTCTTTTATTGTGAGTCAGAGCCTC
AGACTCTAGAGAAGAGAGAAAAGACAGACAATGACATGCTCATGGCTCGTTTTGGC
ATGGCCTAGATGGGCTTTTTGTTTCGTCCTCTCTGTTGTAAGATATCCATGGGTCCCGA
GGACCGGTAGCCCCGGCTGACAAATGCAACTTTGACGCTATTCGCACTGTCGAGAGT
TCACGTTCCTGTACCCAGCCTCTCTCGGAAACTCGCTGACCCCCACCCAGAATGGTTC
CTGTTCAGGGTCTAGTGAGCTGATGGAAGAATGAACTAGAAGGAATCTTTGTGACTC
AGGCAGCAAATTTTCTAGTCCGACGCTCCGCATTGGACCCTTGCCAGCTTGCCTTTGC
TTGCTCTGGTACGCGTAGATACAGTACCTACAGTGCGATTCAACGCCCGCTCAGCTGC
CCCCGCTCTACCTACTGCAGCCATCCATCTCACTCGATTTCCCTTCCTCGCCCATTCTT
GGCTCCGCCTGACACCAGAAACCACCGATTTCATCATCAATCTTACATACTGTCACCT
TACATACCATAGTAGCTTCAGTTTCTATCCTGTCCTTTGCGGCCTGAATCCGAAAACTC
CGTTCTTCTTATTTTCAACTTCGCCTCTCTTCTCATCGCCTTTTGACAATTATATGCCAG
TATCATAACCCAGCAGAAGCCCGCCCCGCTCGTCAAGGTCCCCGACGTCGCCGCTAC
ACAGGCTTCAGGTACAGACAGGCTCTGGTCTGGCTCTTTGTCCCCTCCTGCGTGAGCC
TGGACTGGCAGAGAGCTTGAACCTATAATCAAACCGCGCGGCATCAGTTTCGTCTGC
CTCGATTTCAAATCGTTCTTCACCGTTACGCCTCTTTTCGATCGCCCGTTTCATATTTCG
CGATGACTTTGCCACCCAACTTCCTCTTGACCCCGCAACAGCAGAACCTGCTCTTCGC
TGCTCTCAACTCCAACAAGCAGCAGCTCTCTGGTTCTTCCGTGGACAATTCTCTGACG
GTTTCTCCCACGACGTCCCGCAATTCTGCTGGCCAACAAAAGCCCACCACCGCTACA

GGTTACCAGGAGAGTCCCTTCCTCGACAACTACGACTATGACTTTGGCGATTCTGGTT
TCGACTTCTCCTTCGCCAGCGAGGACCAGCCTTCCATGATTGGAGATCTTCCTGCTGCT
TCCACGGAACCCAACAACAACTCTGGTTCTGTCGCTCTGTCCGACTCCCCAGAGACTG
ATACTCCAGAGAAGCGCAGCTACCCCGACGACGAGGATGACGAAGATAGCCCTGGT
GGGGATCACAAGCGCCGTGAGAGTACCGACAAGGTCCCTAAGAAGCCCGGTCGTAA
GCCTCTTACATCCGAGCCCAGCTCGGTACGTTTTATCAATTGATTACATATAAAATTTA
CTTACTAATCCAAACAATAGAAGCGCAAGGCTCAAAATCGAGCCGCTCAGCGTGCTT
TCAGAGAACGCAAGGAGAAGCATTTGAAGGACCTTGAGACAAAGGTTGAAGAGCTA
GAGAAGGCTTCGCAGGCTGCCAACCATGAGAATGGCATGCTTCGAGCTCAGGTTGAG
CGCATGACAGCTGAACTCAACCAGTATAAGCAGAAGGTCACAGTGATGTCAGCTACC
AAGTCACTGCCCAGAGAAAAGGTTCCTTTCGGCAGCGCTGCCGTCAGCAACCTCGGC
GATGTTAACTTCCAGTTCGAGTTTCCCAAATTTGGCATGCTTCCAGGACCACCTGTCA
GCAAGACTGGCTCATCGCCTACAAGCCCGGATCAGCAAAAGATCACATACCCAAGC
CCTACCAACAGCCTCAACAACAGCGCGCAATCCGCACAACAATTCAAGGATGACTTG
GCCAAGTTCTCAGGTGTCTTCAGCCCTTCCATGGCAAGCTCTGCCACCAACCCCTCTC
GTGCGAGTGTTGACTCTGCAAACTACAGCGTCAATGGCGCTTCCAGCTCCCCATCTGC
TTCATCTCATTCAAACACTGGTCCCAGCTCTTCTTGCGGAACATCGCCTGAGCCTTTCA
ACCAGTCTCCCATGGGCTTCAAGCCCGTTGATACGATGACTACCATTGGCGAGGAGC
AGACATATCAGAACAGCAACAACAACCCCAGTCAATTCGGCAATATTGATCTCAACA
GCACCAACTTTGACTGGCTGTCACAACAGAACGGTGGACAGTTTGACCCACAGCTCT
TTGGTGATTATCGTGAGCCTCAAGAAAACGTATTGGCAAATCCATCTTTCGACGACTT
CTTCAACGATGCTCTTGACAGCGATTTCTTTACTCCTTACAACATGGCTCCTAACAGTC
CCAGCGCGCATCTCAATGGCCAGGCCAAGAAGCCATCGAACCTGATTGATCAGATTG
ATGCTCAAAAGGAGTCCGATGACGAACCTCTCAAGAAGCAGAACATGAACTGTAAT
CAACTGTGGTATGTATCAACTTATAGAATCAGGAAATGTCTGTCTCTAACACGTTTCT
ACAGGGAGAAACTTCAAGCTTGCCCCAAGGCACAGAATGGTGAATTCGACCTCGAC
GGCCTCTGCTCTGAACTTACCAAGAAGGCCAAGTGCTCTGGCACTGGTCCTGTGGTTG
CTGAGACCGACTTTGACACCATTCTGCAAAAGTATATGGGCAAAGACGTCTCCAGTA
GTTGTGTTGCCCAACAGTTGGGTGTGGAAATAAAGTCGAGTGAACCAAAGCAAGATA
AGCATCTTGGTCTGTCGATTGGAGGTGGGGGATCCGGCGGTGGTGGGAGTATGGTGA
GCAAGGGCGAGGAGCTGTTCACCGGGGTGGTGCCCATCCTGGTCGAGCTGGACGGCG
ACGTAAACGGCCACAAGTTCAGCGTGTCCGGCGAGGGCGAGGGCGATGCCACCTAC
GGCAAGCTGACCCTGAAGTTCATCTGCACCACCGGCAAGCTGCCCGTGCCCTGGCCC
ACCCTCGTGACCACCCTGACCTACGGCGTGCAGTGCTTCAGCCGCTACCCCGACCAC
ATGAAGCAGCACGACTTCTTCAAGTCCGCCATGCCCGAAGGCTACGTCCAGGAGCGC
ACCATCTTCTTCAAGGACGACGGCAACTACAAGACCCGCGCCGAGGTGAAGTTCGAG
GGCGACACCCTGGTGAACCGCATCGAGCTGAAGGGCATCGACTTCAAGGAGGACGG
CAACATCCTGGGGCACAAGCTGGAGTACAACTACAACAGCCACAACGTCTATATCAT
GGCCGACAAGCAGAAGAACGGCATCAAGGTGAACTTCAAGATCCGCCACAACATCG
AGGACGGCAGCGTGCAGCTCGCCGACCACTACCAGCAGAACACCCCCATCGGCGAC
GGCCCCGTGCTGCTGCCCGACAACCACTACCTGAGCACCCAGTCCGCCCTGAGCAAA
GACCCCAACGAGAAGCGCGATCACATGGTCCTGCTGGAGTTCGTGACCGCCGCCGGG
ATCACTCTCGGCATGGACGAGCTGTACAAGTGAGACCAGGTTCATGAACAAAGATTC
GGCTGTACACTACAAACTTCGGCGGCATACCCAGCACACAGCTACACAATGATTAAG

GCGTTTACAACAGACAGGATGGGATCGGAAAACCAAGGGATTCCAGCGCTTGATTTA
CCTCCGTCAACGGAATTGGATGCATGGAAAGGAGAAAGGATGATCTATGGTATGATT
ATATAACATGCCATGAAATCTTCGACCTGTTAACTCAGCTTGAAATTTCTTTGGGGATT
TACTTGTGCTTTTTTTTTGGATGGACATTTTTTTGCTGCTATGAATAATTATGACTTATTT
CTAGTTTCAATTGTCAAGGGCTCAACAGGGGATATATGATGATCACTGTGACTTGGCA
ACGCACATGTTGATCAGATATGTACATCAAAGGGACTATTCAACTTTCATTACTACGT
AAAGATGATCAAAGTGTTGGAGGCGCTCTTGTGATACGAGTATTAGTATCCAAGGAT
ATAAATTGCACGCTACAGCAGCAACTTTGAGCCAATCTGCAACATCCAGACCCTGCA
TACGTCAGTAAATTAAGATCTAAAAAAGGTTCAAACGCAAGTAGAAGATCGCCGTG
ACATCATTTTTGGGTCTATCATTGACTGAAAGCTGTTGTGATTTTTTATCATGACATCT
AATCTTAGTCATCAAGTGGAGCCAGATGATCGCGACAAGGAAGCTTTAGCCAGACCA
AACCATCTTGAACAAGTCCTACTCAGCTTACAA

***Yap1*-R-BamHI：** CGCGGATCCCCCACCTCCAATCGACAGACCAAGATGCT

GFP-F-BamHI： CGCGGATCCGGCGGTGGTGGGAGTATGGTGAGCAAGGGCGAGGA

GFP-R： CTTTGTTCATGAACCTGGTCTCACTTGTACAGCTCGTCCATGC

3UTR-F： GCATGGACGAGCTGTACAAGTGAGACCAGGTTCATGAACAAAG

Yap1 sequence in yellow font, 5UTR and 3UTR in blue font at both ends, termination codon in red font, GFP sequence in green font and Linker sequence in pink font.

Appendix SD: Sequencing results for backfill

1.Backfill sequence：

CGGGAAAGGAGGAAATGGCCGCTTTTCTGGATTCATCGACTGTGGCCGGCTGGGTGT
GGCGGACCGCTATCAGGACATAGCGTTGGCTACCCGTGATATTGCTGAAGAGCTTGG
CGGCGAATGGGCTGACCGCTTCCTCGTGCTTTACGGTATCGCCGCTCCCGATTCGCAG
CGCATCGCCTTCTATCGCCTTCTTGACGAGTTCTTCTGAGGTACCAAGTACTTTCGCAT
CCAGTGAGCTGTTTTCTGGCAGCCGTTGAGATACGAAATTACGGTTGAAATTTCAGCG
ATTGATAACTGGACTTGACCCATTGGGAAGAATAATTGCCATTGATTGGTCGAAAGT
GAGGTGAATGCGCCGTGTGGCAAACTCCGAATCTTGGTCTAAATCCGGGGGCGGGGA
GAACTTCGACAGGACCGCGTGGTTGTGGCTCGGGGATAAGGCAATGGCTGCAGATGA
GGTGAACGGGGCATTGTTAGCAGTCAGAAACATTA

2.NEO Sequence:

GGTCTTGGGGTGGAGAGGCTATTCGGCTATGACTGGGCACAACAGACAATCGGCTGC
TCTGATGCCGCCGTGTTCCGGCTGTCAGCGCAGGGGCGCCCGGTTCTTTTTGTCAAGA
CCGACCTGTCCGGTGCCCTGAATGAACTGCAAGACGAGGCAGCGCGGCTATCGTGGC
TGGCCACGACGGGCGTTCCTTGCGCAGCTGTGCTCGACGTTGTCACTGAAGCGGGAA
GGGACTGGCTGCTATTGGGCGAAGTGCCGGGGCAGGATCTCCTGTCATCTCACCTTGC
TCCTGCCGAGAAAGTATCCATCATGGCTGATGCAATGCGGCGGCTGCATACGCTTGA
TCCGGCTACCTGCCCATTCGACCACCAAGCGAAACATCGCATCGAGCGAGCACGTAC
TCGGATGGAAGCCGGTCTTGTCGATCAGGATGATCTGGACGAAGAGCATCAGGGGCT
CGCGCCAGCCGAACTGTTCGCCAGGCTCAAGGCGAGCATGCCCGACGGCGAGGATCT
CGTCGTGACCCATGGCGATGCCTGCTTGCCGAATATCATGGTGGAAAATGGCCGCTTT
TCTGGATTCATCGACTGTGGCCGGCTGGGTGTGGCGGACCGCTATCAGGACATAGCG
TTGGCTACCCGTGATATTGCTGAAGAGCTTGGCGGCGAATGGGCTGACCGCTTCCTCG

TGCTTTACGGTATCGCCGCTCCCGATTCGCAGCGCATCGCCTTCTATCGCCTTCTTGAC
GAGTTCTTCTGGAGAGACCCA

3.GFP partial sequence：
AGGCTTCTCTTCAGCTCGAGCTGCCCTGAATGGGTGCGACTTCTGTCACGGCCGGCCT
GCCCCAATGCTACGTCCGGGAGCGCACGTCTTCTTCTGGACGACGGGAACTACAACA
CCCGCGCCGAGGCGAAGTTCGAGGGCCACACCCCGGTGAACCGCATATGCCTGAAC
GGGATCAACTTCCCCGAGGACGGCCAATTCCTGGGGCACAAGCTGGAGTACAACTAC
AGCCGCCGCCACGTATTTCTCTTGGGGGGGGAGGGAAAGGGAACGCATAA

Appendix SE: Information about the Fusarium strain Error! Reference source not found.

Table S3. Primers, GenBank accession numbers and amplification procedures used for strain identification

Sequence (accession)	Primer Sequence（5′ to 3′）	Amplification Program
ITS (OP412776)	ITS1: TCC GTA GGT GAA CCT GCG G<br>ITS4: TCC TCC GCT TAT TGA TAT GC	Started with an initial denaturation at 95 °C for 15 s followed by 30 cycles of 15 s at 95 °C, 15 s at 57 °C and 1min at 72 °C with a final extension step of 5 min at 72 °C.
TEF-1α (OP330068)	EF-1: ATG GGT AAG GAA GA CAA GAC<br>EF-2: GGA AGT ACC AGT GAT CAT GTT	Started with an initial denaturation at 95 °C for 5 min, followed by 30 cycles of 45 s at 94 °C, 40 s at 58 °C, and 1 min at 72 °C with a final extension of 10 min at 72 °C.
TUB (OP382711)	TUB-2Fd: GTB CAC CTY CAR ACC GGY CAR TG<br>TUB4RD: CCR GAY TGR CCR AAR ACR AAG TTG TC	Started with an initial denaturation at 94 °C for 5 min, followed by 30 cycles of 30 s at 94 °C, 30 s at 52 °C and 30 s at 72 °C with a final elongation of 7 min at 72 °C.

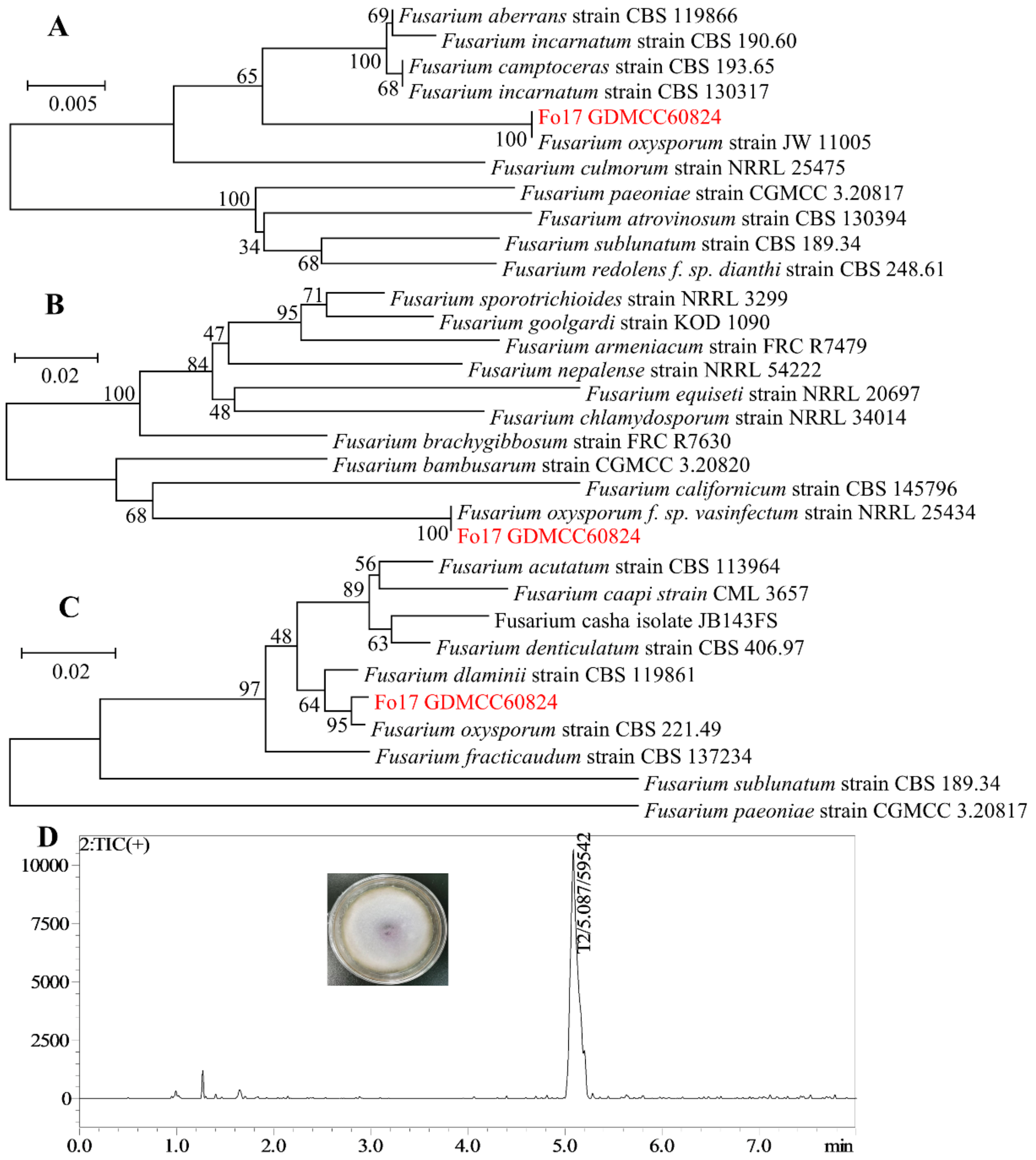

Figure S1. Neighbor-joining tree constructed based on the internal transcribed spacer (OP412776, A), translation elongation factor 1-alpha (OP330068, B) and β-tubulin (OP382711, C) sequences. Total ions chromatogram for T-2 toxin produced by Fusarium oxysporum (Fo17, GDMCC60824) (D).
